# Echocardiographic right ventricular remodeling after percutaneous atrial septal defect closure

**DOI:** 10.1016/j.ijcchd.2023.100459

**Published:** 2023-04-19

**Authors:** Daan Bosshardt, Michiel Voskuil, Gregor J. Krings, Mirella M.C. Molenschot, Maarten J. Suttorp, Heleen B. van der Zwaan, Martijn C. Post

**Affiliations:** a Department of Cardiology, University Medical Center Utrecht, Utrecht, the Netherlands; b Department of Cardiology, St. Antonius Hospital, Nieuwegein, Utrecht, the Netherlands

**Keywords:** Secundum atrial septal defects, Percutaneous closure, Right ventricle, Remodeling

## Abstract

**Background:**

In order to prevent right ventricular (RV) dysfunction, closure of secundum type atrial septal defects (ASD) is often indicated and percutaneous closure is the preferred treatment modality to do so. The magnitude and time course of RV remodeling is still incompletely understood.

**Methods:**

This retrospective cohort study included consecutive patients who underwent percutaneous secundum ASD closure in two tertiary referral centers in The Netherlands. Main study parameters were RV and right atrial dimensions measured with transthoracic echocardiography before and after percutaneous ASD closure. Secondary outcome was change in New York Heart Association (NYHA) functional class at follow-up.

**Results:**

From the 454 patients who underwent secundum ASD closure, 88 patients (median age 46 [range 17–84]) were included. The majority of RV and right atrial dimensional improvement occurred within 24 h. After a median follow-up of 569 days (IQR: 280–772) a further decrease in dimensions was observed. Comparing baseline and latest follow-up, end-diastolic RV basal diameter decreased from 4.5 SEM 0.1 to 3.9 SEM 0.1 cm (*p* < *0.001)* and end-systolic right atrial area from 22.9 SEM 1.0 to 17.9 SEM 0.7 cm^2^ (*p* < *0.001)*. No significant changes in RV function were observed. NYHA functional class improved from 1.5 at baseline (IQR: 1.0–2.0) to 1.0 (IQR: 1.0–1.5) at latest follow-up (*p* < *0.001)*.

**Conclusion:**

Remodeling of the RV heart dimensions commences within 24 h after percutaneous secundum ASD closure for the majority of patients, followed by a further gradual recovery. A concurrent improvement of NYHA functional class was observed during follow-up.

## 1 Introduction

Atrial septal defects (ASDs) are a common form of congenital heart defects accounting for approximately 13% of all congenital heart defects and for 30–40% of congenital defects diagnosed in adulthood [1,2]. Seventy percent of all ASDs concern the secundum type.

A significant left-to-right shunt (L-R-shunt) on the level of the right atrium (RA) may lead to right ventricular (RV) volume overload [3]. RV overload may cause atrial arrhythmias, exercise intolerance, fatigue, dyspnea, pulmonary hypertension, tricuspid valve insufficiency and eventually overt heart failure [4].

Closure of the secundum ASD is indicated in patients with a hemodynamic significant shunt, in other words, with RV overload in the absence of pulmonary arterial hypertension and should be considered in patients with suspicion of a paradoxical embolism [3]. A percutaneous approach for secundum ASD closure is the method of choice in over eighty percent of cases, in which a percutaneous approach is technically feasible [3]. With a major complication rate ≤1% (including retroperitoneal bleeding, air embolism, device embolism and aortic erosion) percutaneous closure is superior to surgical closure concerning complication rate and hospital stay duration [[5], [6], [7]].

Anatomical and functional evaluation of the RV is critical for identifying a L-R shunt and evaluating RV volume overload. While cardiac magnetic resonance imaging (CMR) is the gold standard for the assessment of RV morphology and RV ejection fraction as it allows for a time resolved three dimensional evaluation of the RV, in clinical practice CMR is not routinely acquired in the diagnostic work-up for secundum ASD closure [3, 8]. Instead, echocardiography is the first-line diagnostic technique that allows both the size and function of the RV to be sufficiently evaluated in a single examination [3].

The effect of percutaneous ASD closure on RV remodeling is still incompletely understood. The aim of the current study is to describe changes in RV size and function after percutaneous ASD closure using transthoracic echocardiography (TTE).

## 2 Methods

### 2.1 Patient and procedural characteristics

This retrospective study was conducted in patients with a secundum ASD, who underwent successful percutaneous closure between November 1996 and January 2018 at the St. Antonius Hospital Nieuwegein or University Medical Center Utrecht, The Netherlands. Patients were eligible if a TTE was available before, within one day after and at least six months after percutaneous closure. Baseline characteristics, New York Heart Association (NYHA) functional class, as well as indication for closure, type and size of device implanted, procedural success rate and peri- and post procedural complications were reviewed from the electronic patient records. When multiple devices were implanted, the device with the highest diameter was used for analysis. Both local ethical commissions approved the study and waived the necessity of informed consent.

### 2.2 Follow-up

Data about residual shunting, complications and change in NYHA functional class were collected up until January 1, 2019, with a median follow-up duration of 569 days (IQR: 280–772).

### 2.3 Transthoracic echocardiography

**Fig. 1:**
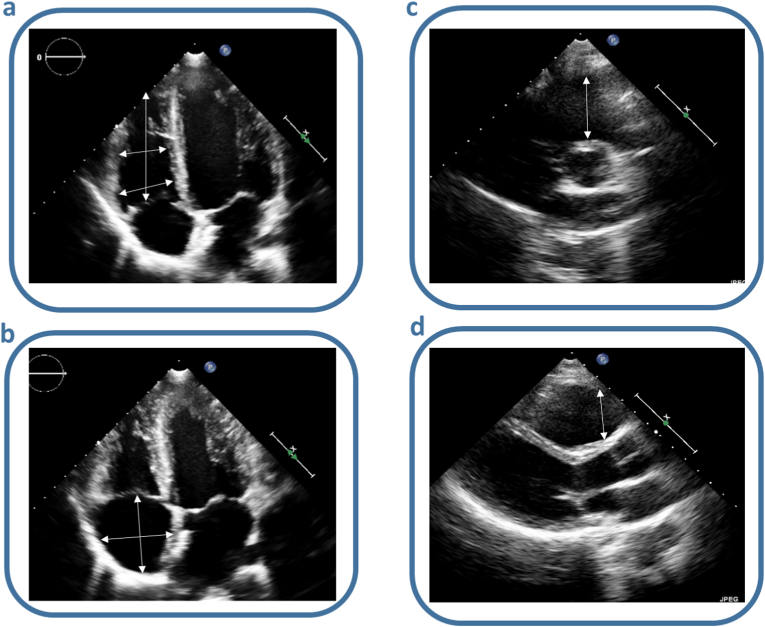
Overview of the methodology used for RV dimensions measurements: in the apical 4-chamber view, the end diastolic basal, mid a longitudinal RV a), end-systolic major and minor RA b) and the end-diastolic proximal right ventricular outflow tract on the parasternal short c) and long axis d).

Data from TTE were collected from all patients pre-, one-day post-procedure and at follow-up with a minimal follow-up duration of 6 months and a maximum of three years after closure. Data were obtained by performing measurements in the original saved echocardiographic images in Xcelera or IntelliSpace Cardiovascular System (Philips, Best, the Netherlands). Measurements and interpretations were made according to the guidelines by Rudski et al. [9]. RV minor and longitudinal diameters were obtained from the apical 4-chamber view as well as the RA major and minor diameter, the RA area, tricuspid annular plane systolic excursion (TAPSE) and RV fractional area change (FAC). The proximal RV outflow tract (RVOT) was measured in the parasternal long axis (PLAX) and in the parasternal short axis (PSAX) at aortic valve level. An example of the windows and measurements techniques is provided in Fig. 1. Inferior vena cava diameter was obtained from the subcostal view. Pulmonary systolic pressure/RV systolic pressure (RVSP) was estimated using the modified Bernoulli equation [9]. If TAPSE was not available but the report mentioned a good RV function, TAPSE was estimated to be 2.3 mm, which is the mean value of TAPSE in a healthy population as previously described [9].

### 2.4 Statistical analysis

Statistical analysis was performed using IBM SPSS Statistics for Windows, Version 26.0 (Armonk, NY: IBM Corp.). Quantitative data were visually analyzed for normality using Q-Q plots and histograms. Data were described as mean ± standard deviation (SD), median with interquartile range (IQR) or absolute number (percentage) as deemed appropriate. Mean ± standard error of the mean (SEM) was reported for parameters with imputed data. Comparisons of continuous parameters between baseline and follow-up were performed using the paired *t*-test or the Wilcoxon sign rank test, depending on data distribution. Independent predictors for change in RV and RA parameters were analyzed with (multivariable) linear regression. Multiple Imputation by Chained Equations (MICE) package in R version 4.0.3 (R Core Team, 2014) using 40 imputations and seed = 5000 was used to impute missing TTE values [10]. Probability (p) values of <0.05 were considered statistically significant.

## 3 Results

### 3.1 Patient characteristics and complications

In total, 454 consecutive patients were scheduled for percutaneous ASD closure between November 1996 and December 2017. Of those, echocardiographic reports and/or images were missing in 359 patients. Seven patients did not undergo percutaneous closure because the secundum ASD turned out to be unsuitable. The baseline characteristics of the 88 included patients are presented in Table 1. Mean age at time of procedure was 46 SD 16 years with a male: female ratio of 2:3. Periprocedural data are displayed in Table 2. RV overloading was the indication for closure in most patients (72.7%), followed by cryptogenic ischemic cerebrovascular accident (iCVA) or transient ischemic attack (23.8%).

**Table 1:** Baseline patient characteristics.

	All included patients (n = 88)
Age (years)	46 (17–84)
Male	35 (39.7%)
Length (cm)	173 SD 9
Weight (kg)	77 SD 17
Systolic blood pressure (mmHg)	127 SD 18
Diastolic blood pressure (mmHg)	79 SD 11
History	
CVA/TIA	11 (12.5%)/14 (15.9%)
PAD/CAD	1 (1.1%)/3 (3.4%)
Hypertension	22 (25.0%)
Supraventricular tachycardia	18 (20.5%)
Smoker	12 (13.6%)
Hypercholesterolemia	10 (11.3%)
Diabetes mellitus	4 (5.7%)
ECG	
Sinus	82 (93.2%)
Pacemaker	1 (1.1%)
Atrial fibrillation	5 (5.6%)
RBBB	9 (10.2%)

Values are expressed as mean ± standard deviation, median (range) or n (%).

Abbreviations: SD: Standard Deviation, CVA: cerebrovascular incident, TIA: transient ischemic attack PAD: peripheral artery disease, CAD: coronary artery disease, RBBB: right bundle branch blockade.

**Table 2:** (Peri) procedural data of all included patients.

	All included patients (n = 88)
Indication closure	
RV overloading	64 (72.7%)
CVA or TIA	21 (23.8%)
Prevention of decompression sickness	2 (2.3%)
Simultaneous venous and arterial embolus	1 (1.1%)
Device information	
Diameter device (mm)	24 (11–40)
Amplatzer Septal Occluder	38 (43.1%)
Occlutech Occluder 2	26 (29.5%)
Occlutech Occluder	19 (21.6%)
Ceraflex	5 (5.7%)
Hospital stay (days)	2 (2–4)

Values are expressed as median (range) or n (%).

Abbreviations: ASD: type 2, CVA: cerebrovascular incident, TIA: transient ischemic attack, AF: atrial fibrillation.

Periprocedural complications were one device dislocation, one cardiac tamponade and one secondary surgical closure one day postprocedural due to residual shunting, all of which all were successfully treated without further sequelae. Three patients (3.4%) developed new onset atrial fibrillation periprocedurally, one of which evolved into persistent atrial fibrillation. After a median follow-up of 569 days (IQR: 280–772), a (residual) L-R shunt on color Doppler was present in six patients (6.8%), one of which was significant and required surgical closure three years after the procedure. In another patient, surgical closure was performed after three years because the device protruded into the aorta. Four patients died during follow-up (4.5%): one due to sepsis, one of pulmonary fibrosis and two of an unknown cause. Two patients (2.3%) suffered from an iCVA during follow-up, one of which was a 73-year-old female with a high cardiovascular risk profile and the other a 51-year-old female without known cardiovascular risk factors. Neither patient suffered from AF nor had an intracardiac thrombus. However, echocardiography in the latter patient did show a grade 2 right-to-left shunt, when performing the Valsalva maneuver.

When comparing functional outcome for the entire cohort using the NYHA functional class prior to closure and during follow-up, a significant improvement was observed from 1.5 (IQR: 1.0–2.0) to 1.0 (IQR: 1.0–1.5), *p* < *0.001*.

### 3.2 Transthoracic echocardiography

An overview of TTE parameters at baseline, one-day post procedure and during latest follow-up are displayed in Table 3. A dedicated RV view was available for 57 (22%) of the examinations.

**Table 3:** Right atrial and ventricular dimensions and function in all patients (n = 88), median follow-up 569 days (IQR: 280–772).

	Pre-closure	One day post procedure	Follow-up	*P*- value one day	p-value pre vs. follow-up	p-value 1 day vs. follow-up
***End-diastolic***						
***RV basal width (cm)***	4.52 SEM 0.10	4.33 SEM 0.08	3.94 SEM 0.07	0.034	<0.001	<0.001
***RV mid-width (cm)***	3.96 SEM 0.11	3.75 SEM 0.09	3.35 SEM 0.08	0.087	<0.001	<0.001
***RV longitudinal length (cm)***	8.24 SEM 0.11	8.02 SEM 0.13	7.90 SEM 0.11	0.088	0.006	0.355
***Prox RVOT PLAX (cm)***	3.78 SEM 0.08	3.56 SEM 0.07	3.25 SEM 0.06	0.003	<0.001	<0.001
***RVOT PSAX (cm)***	3.73 SEM 0.08	3.46 SEM 0.07	3.31 SEM 0.07	0.006	<0.001	0.067
***RAA (cm***^***2***^***)***	17.20 SEM 1.01	15.25 SEM 0.83	13.94 SEM 0.75	0.001	<0.001	0.011
***RA major (cm)***	4.28 SEM 0.14	4.11 SEM 0.11	4.02 SEM 0.11	0.065	0.005	0.345
***RA minor (cm)***	4.06 SEM 0.10	3.67 SEM 0.09	3.58 SEM 0.08	<0.001	<0.001	0.239
**End-systolic**						
***RV basal width (cm)***	3.63 SEM 0.09	3.49 SEM 0.08	3.23 SEM 0.07	0.120	<0.001	0.003
***RV mid-width (cm)***	3.01 SEM 0.09	2.98 SEM 0.08	2.63 SEM 0.07	0.782	<0.001	<0.001
***RV longitudinal length (cm)***	6.64 SEM 0.09	6.37 SEM 0.10	6.60 SEM 0.11	0.011	0.752	0.066
***RAA (cm***^***2***^***)***	22.91 SEM 0.95	20.26 SEM 0.85	17.94 SEM 0.74	<0.001	<0.001	<0.001
***RA major (cm)***	5.44 SEM 0.13	5.23 SEM 0.12	4.89 SEM 0.10	0.024	<0.001	<0.001
***RA minor (cm)***	4.54 SEM 0.11	3.92 SEM 0.09	3.86 SEM 0.08	<0.001	<0.001	0.440
***RV function***						
**TAPSE (cm)**	2.47 SEM 0.07	2.32 SEM 0.04	2.36 SEM 0.05	0.046	0.134	0.558
**RV FAC (%)**	37.73 SEM 2.49	35.80 SEM 1.65	34.09 SEM 1.70	0.506	0.244	0.455
eRVSP* (n = 35)	32.00 SD 2.04	NA	27.8 SD 1.89	NA	0.005	NA

Abbreviations: RV: right ventricle, prox RVOT: proximal right ventricular outflow tract, RAA: right atrium area, TAPSE: tricuspid annular plane systolic excursion, FAC: fractional area change, eRVSP estimated right ventricular systolic pressure, SEM: standard error of the mean, SD: standard deviation, NA: not available * eRVSP was available only for a subgroup of patients (n = 35).

**Fig. 2:**
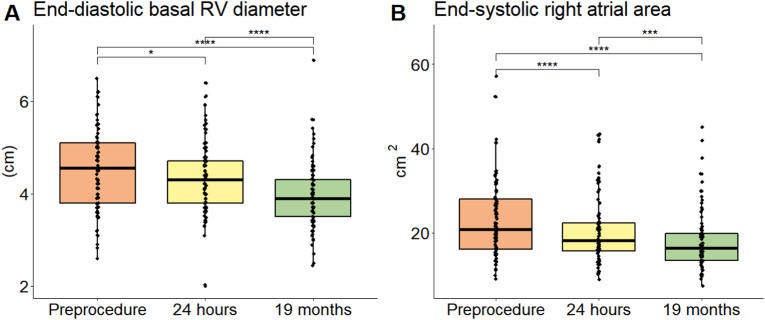
Improvement in echocardiographic end-diastolic right ventricular (RV) basal diameter before and after secundum atrial septal defect closure A) and in end-systolic right atrial area B) *p < 0.05, ***p < *0.001.*

The majority of RV dimensional parameters decreased within one day after closure in 50 patients (57%), with a gradual improvement during follow-up in 60 patients (68%). At follow-up RV dimensions were normalized in 23 (26%) of the patients at follow-up. In particular, mean end-diastolic RV basal diameter decreased after one day (4.5 SEM 0.1 to 4.3 SEM 0.08 cm, *p* = *0.034),* and at follow-up (3.9 SEM 0.07 cm, *p* < *0.001* for differences with one day follow-up). (Fig. 2a).

RA dimensions also decreased significantly: end-systolic RA major dimensions decreased after one day (5.4 SEM 0.1 to 5.2 SEM 0.1 cm, *p* = *0.024*) with a further decrease at follow-up (4.9 SEM 0.1 cm, *p* = < *0.001* versus one-day follow-up). End-systolic RA area too decreased after one day (22.9 SEM 1.0 to 20.3 SEM 0.9 cm^2^, *p* < 0.001) and at follow-up (17.9 SEM 0.7 cm^2^ at follow-up, p < 0.001 versus one day after the procedure) (Fig. 2b).

Concerning RV function parameters, a small, but statistically significant decrease in TAPSE was seen between baseline and one day post procedure (2.47 SEM 0.07 to 2.32 SEM 0.04 cm, *p* = *0.046*). However, comparing baseline and follow-up TAPSE, no differences were observed (2.47 ± 0.07 to 2.36 SEM 0.05 cm, at follow-up, *p* = *0.134* versus baseline). No significant changes in RV FAC were observed comparing baseline and one day post-procedure (37.7% SEM 2.5%, vs 35.8% SEM 1.7%, *p* = *0.506),* and follow-up (34 SEM 1.70%, *p* = *0.455* compared to one day follow-up). Estimated RVSP could only be obtained for a subgroup of patients (n = 35). In this subgroup estimated RVSP was significantly lower during follow-up compared to baseline (32.0 SEM 2.0 to 27.8 SEM 1.9 mmHg, *p* = *0.005*).

No predictors for improvement of RV and RA parameters could be identified in univariate and multivariable analysis. Variables entered in the model were age, sex, indication for closure, weight, length, systolic and diastolic blood pressure, hypertension hypercholesterolemia, diabetes, coronary/peripheral artery disease, smoking, supraventricular tachycardia, indication closure, NYHA functional class, EKG rhythm, EKG conduction, device type and device diameter.

## 4 Discussion

The main findings of our study were: 1) A significant improvement in RV and RA parameters within the first 24 h after percutaneous secundum ASD closure in the majority of patients, followed by 2) a late decrease in RV and RA dimensions at follow-up, with complete normalization of RV dimensions in a subgroup of patients and 3) a low periprocedural complication rate at our institutions.

This initial rapid improvement is thought to be the direct consequence of the fast decrease in RA and RV preload after eliminating the volume overloading on the right side of the heart. Similar results were found in previous studies [[11], [12], [13], [14], [15]].

According to previous literature, following abrupt decrease in RV and RA dimensions after closure, a further gradual volumetric decrease resulting in normalization takes place within the next six to twelve months [11,[16], [17], [18], [19]]. In our study, mean RV and RA dimensions significantly improved after closure, with complete normalization only for a subgroup of patients. This finding of incomplete remodeling is consistent with previous results, in which persistent right ventricular enlargement was found in approximately two-thirds of patients after ASD closure [12,[20], [21], [22]] and could possibly be explained by the fact that the mean age in our case series is relatively old. It has been shown that age at the time of closure was inversely related to the occurrence of reverse remodeling and that closing an ASD at an age above 60 improved RV dimensions but did not result in normalization of RV dimensions [20,22]. This might be explained by the fact that elderly patients have been exposed to chronic volume overloading for a longer period and thus have a reduced capacity for reverse remodeling. Furthermore, a similar process of incomplete inverse RV remodeling is observed in tetralogy of Fallot patients who have undergone pulmonary valve replacement after a prolonged period of RV volume overload due to pulmonary valve insufficiency [23].

In the current study, we acquired RV FAC and TAPSE for the evaluation of RV function. RV FAC is a measurement of RV systolic function and showed a good correlation with RV ejection fraction on cardiac magnetic resonance imaging and decreased RV FAC has been associated with increased risk of all-cause mortality [[24], [25], [26]]. TAPSE, as measurement for longitudinal RV function, is correlated with global RV function [27]. We found a trend toward decrease in both RV FAC and TAPSE immediately after percutaneous closure. This is in line with previous work from Vitarelli et al. and Akula et al. who found a significant decrease in TAPSE after percutaneous closure [16,18,28]. They postulated that the increased preload prior to closure results in a high basal RV systolic function and thus results in high values for load dependent parameters such as TAPSE. These functional parameters decrease after closure, due to a decreased preload. Thus, rather than loss of RV function, this decrease in functional parameters seems to reflect normalization of normal RV geometry [18]. Furthermore, it seems this decrease in RV functional parameters does not reflect a worsening in functional outcomes. On the contrary, in our study NYHA functional class had improved at follow-up compared to the pre-procedural functional status.

Several studies also found NYHA functional class improvement after percutaneous secundum ASD closure [15,17,22,29,30]. However due to data availability we were not able to investigate if this subjectively measured improvement is reflected by an improvement in objectively measured function, i.e., cardiopulmonary exercise testing. Improvement of peakVO2 after percutaneous closure has been extensively described and is found to occur simultaneously with NYHA functional class improvement [[31], [32], [33], [34], [35]]. Transient improvement in peakVO2 is even observed in asymptomatic patients undergoing percutaneous closure, which emphasizes the benefit of ASD closure even in this patient group [36]. Interestingly, improvement in NYHA functional class and peakVO2 seems to occur only after a minimum duration of six months after percutaneous closure [[31], [32], [33], [34]]. Unfortunately, in the current study, NYHA functional class was not evaluated until at least 6 months after the procedure, meaning that any changes in functional class in the first months after closure could not be examined.

While parameters such as increased age and elevated RVSP have previously been associated with the amount of reverse remodeling, in our current study these and other baseline parameters were no predictors for reverse remodeling [37,38]. A possible explanation is the relatively small size of our study population, resulting in insufficient power or relatively old age of the population in our study, which might have eliminated age as predictor. Additionally, RVSP could be determined only in a small subgroup of patients and could therefore not be entered in the regression analysis.

A meta-analysis of 23 studies describing outcomes after percutaneous secundum ASD closure in adults report a mean success rate of 96.7%, with a range from 78.7% to 100% [30]. Our success rate was similar, with only one patient requiring immediate secondary surgical closure after a failed percutaneous attempt. In total, including follow-up, secondary surgical closure was required in two percent of our patients, which is comparable to the 1.2% described in their study.

The association between percutaneous ASD closure and atrial arrhythmias is controversial. On the one hand, reverse atrial remodeling after closure might lead to a decreased chance of supraventricular arrhythmias [39]. On the other hand, the presence of a closure device has a possible pro-arrhythmogenic effect [40]. Moreover, after percutaneous closure, transseptal punctures for instrumentation of the left atrium during percutaneous catheter ablation are challenging for the operator because of the presence of the device [41]. A recent meta-analysis of 25 studies that reported the prevalence of atrial arrhythmia before and after closure of an ASD in the atrium could not demonstrate an association between closure of an ASD and reduction of atrial arrhythmia, but did find an association between reduction in atrial arrhythmia in a subgroup of patients ≥40 years of age [42]. However, the incidence of new arrhythmia after closure in our cohort was 3.4%, and incidence rates of up to 5.8% are described in the literature [30]. Thus, although improvement in arrhythmic burden may be observed in a specific subgroup of AF patients after percutaneous ASD closure, no general conclusion on the effects on the incidence of atrial arrhythmia can be drawn based on the current literature.

## 5 Limitations

Various limitations apply to the current study. The most important one being the limited availability of TTE images from referred patients, resulting in the exclusion of a large number of patients. This was due to the retrospective nature of our study, as we were confronted with a relatively high frequency of missing data, as not all the desired ultrasound windows and measurements were available. Listwise deletion of missing echocardiographic values of our 88 study subjects would have resulted in a loss of power and would have been a possible source for selection bias. To prevent this, we used multiple imputation to increase power and reliability of our analysis [43]. Furthermore, only one- and two-dimensional echocardiographic RV measurements were used because these have been routinely applied over the last 20 years. RV imaging is thereby limited by the complex multipartite, multiplanar morphology of the RV [44]. 3D echocardiography or CMR data more accurately assess RV volumes and ejection fraction [45,46]. Furthermore, our real world data show that the dedicated RV view required in the guidelines has not yet been fully implemented in clinical practice, as it was only available in 22% of the examinations in our study [9]. Although a correlation study comparing RV dimensions and function in the standard apical 4-chamber view versus MRI demonstrated a moderate to strong correlation between the two modalities in several parameters, the use of the standard view may have underestimated RV size and function. This is because dedicated RV view measurements are typically higher and more reproducible than those obtained from the standard view [47,48]. Thus, our data stress the importance of increased awareness of the dedicated RV view for RV dimensions analysis in clinical practice.

## 6 Conclusions and future perspectives

In this retrospective study of patients who underwent percutaneous secundum ASD closure, we found that in the majority of patients, reverse remodeling of the RV and RA commences within 24 h after closure with a further gradual improvement of right heart dimensions 19 months later and a concurrent improvement in NYHA functional class. Normalization of dimensions was found in 26% of patients. We found a high success- and low complication rate of percutaneous closure. Larger (prospective) studies should confirm the concept of early percutaneous closure, to maximize reverse remodeling and improve clinical outcome.

## Data availability

The datasets analyzed during the current study will become available from the corresponding author on reasonable non-commercial request.

## Declaration of competing interest

The authors declare that they have no known competing financial interests or personal relationships that could have appeared to influence the work reported in this paper.
